# Metabolic engineering strategy for synthetizing *trans*-4-hydroxy-l-proline in microorganisms

**DOI:** 10.1186/s12934-021-01579-2

**Published:** 2021-04-21

**Authors:** Zhenyu Zhang, Pengfu Liu, Weike Su, Huawei Zhang, Wenqian Xu, Xiaohe Chu

**Affiliations:** 1 Collaborative Innovation Center of Yangtze River Delta Region Green Pharmaceuticals, Zhejiang University of Technology, Hangzhou, 310014 Zhejiang People’s Republic of China; 2 School of Pharmaceutical Sciences, Zhejiang University of Technology, Hangzhou, 310014 Zhejiang People’s Republic of China

**Keywords:** *Trans*-4-hydroxy-l-proline, Biosynthetic pathway, Proline hydroxylases, Metabolic engineering

## Abstract

*Trans-*4-hydroxy-l-proline is an important amino acid that is widely used in medicinal and industrial applications, particularly as a valuable chiral building block for the organic synthesis of pharmaceuticals. Traditionally, *trans-*4-hydroxy-l-proline is produced by the acidic hydrolysis of collagen, but this process has serious drawbacks, such as low productivity, a complex process and heavy environmental pollution. Presently, *trans-*4-hydroxy-l-proline is mainly produced via fermentative production by microorganisms. Some recently published advances in metabolic engineering have been used to effectively construct microbial cell factories that have improved the *trans-*4-hydroxy-l-proline biosynthetic pathway. To probe the potential of microorganisms for *trans*-4-hydroxy-l-proline production, new strategies and tools must be proposed. In this review, we provide a comprehensive understanding of *trans*-4-hydroxy-l-proline, including its biosynthetic pathway, proline hydroxylases and production by metabolic engineering, with a focus on improving its production.

## Background

**Fig. 1:**
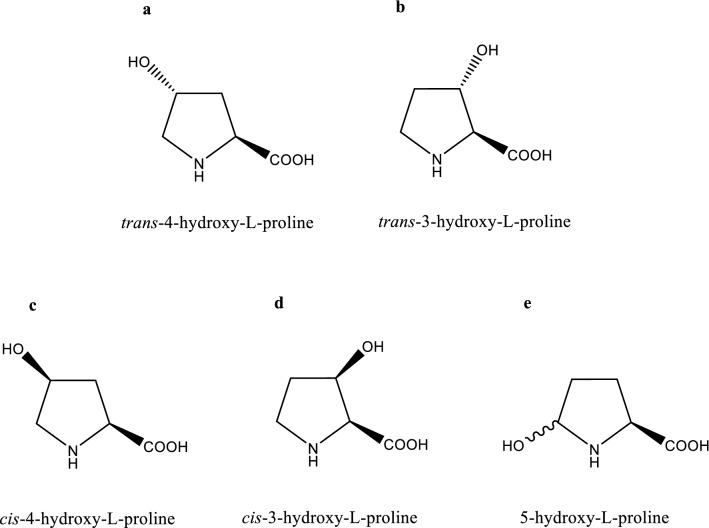
Structures of the hydroxyproline isomers. **a** trans-4-hydroxy-l-proline. **b** trans-3-hydroxy-l-proline. **c** cis-4-hydroxy-l-proline. **d** cis-3-hydroxy-l-proline, and **e** 5-hydroxy-l-proline

**Fig. 2:**
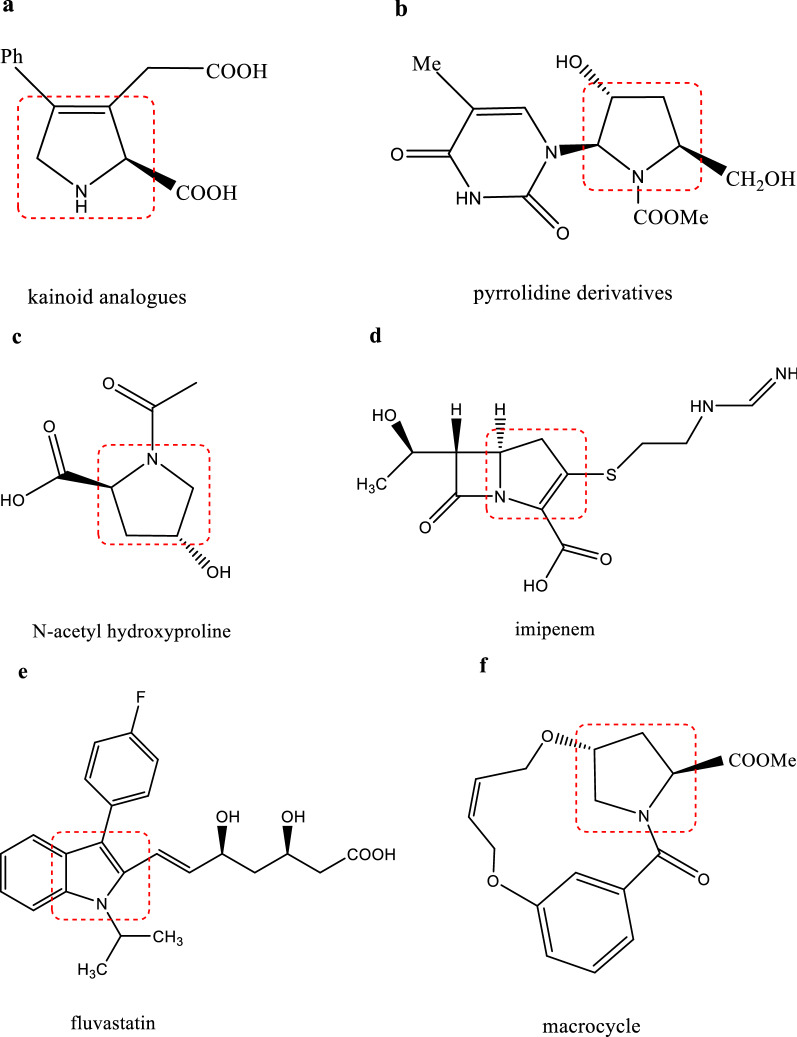
Compounds for which the parent structure is hydroxyproline

Hydroxyprolines are a series of hydroxylated derivatives of proline comprising six isomers—*trans*-4-hydroxy-l-proline (*t*4Hyp), *trans*-3-hydroxy-l-proline (*t*3Hyp), *cis*-4-hydroxy-l-proline (*c*4Hyp), *cis*-3-hydroxy-l-proline (*c*3Hyp), *trans*-5-hydroxy-l-proline (*t*5Hyp), and *cis*-5-hydroxy-l-proline (*c*5Hyp) (Fig. 1). Furthermore, *t*4Hyp is a major amino acid in collagen proteins, which contain two α1 and one α2 polypeptide chain, to allow the sharp twisting of the collagen helix and are the major extracellular components of connective tissues, such as skin, tendon, cartilage, blood vessels, and bone. The helical region of collagen comprises Gly-X-Y repeats, with *t*4Hyp being present only in the Y position [1]. *T*4Hyp was previously considered to have little nutritional significance but is now thought to be a major precursor for the synthesis of glycine, pyruvate, and glucose. Some evidence supports the ability of *t*4Hyp to scavenge oxidants, regulate the state of cellular reduction and stimulate the expression of anti-oxidative enzymes in the cell [2, 3]. *T*4Hyp is required for normal secretion after protein synthesis and contributes to the integrity of the triple-helical conformation [4]. In animals, *t*4Hyp is produced by the posttranslational hydroxylation of proline residues in proteins [5, 6]. *T*4Hyp is a nonessential α-amino acid and is present in several secondary metabolites (e.g., echinocandins and etamycin) [7]. Additionally, *t*4Hyp has been widely used in the medicine, biochemistry, food, and cosmetic industries and is usually used as a valuable building block for the synthesis of many pharmaceuticals [8]. *T*4Hyp can be used to synthesize many compounds, such as glutamic acid analogs, kainoid analogs (a class of nonproteinogenic amino acids with a broad spectrum of biological activities), arginine analogs, and pipecolic acid and its derivatives (which play important biological roles as components of peptides, proteins, and intermediates for the synthesis of conformationally constrained molecular scaffolds) through diastereoselective synthesis, *N*,*N*ʹ-dioxides, carbapenem antibiotics (imipenem, meropenem, panipenem), angiotensin-converting enzyme inhibitors, *N*-aryl pyrroles (important organic compounds for medicinal and material sciences), oxaceprol (an atypical inhibitor of inflammation used for the treatment of conditions affecting the connective tissues, such as osteoarthritis), oligomers, and macrocycles (usually used as valuable sources of bioactive molecules that have properties such as good solubility and metabolite stability for drug discovery) (Fig. 2) [9–18].

## Artificial biosynthetic pathway of trans-4-hydroxy-l-proline in *Escherichia coli* and *Corynebacterium glutamicum*

**Fig. 3:**
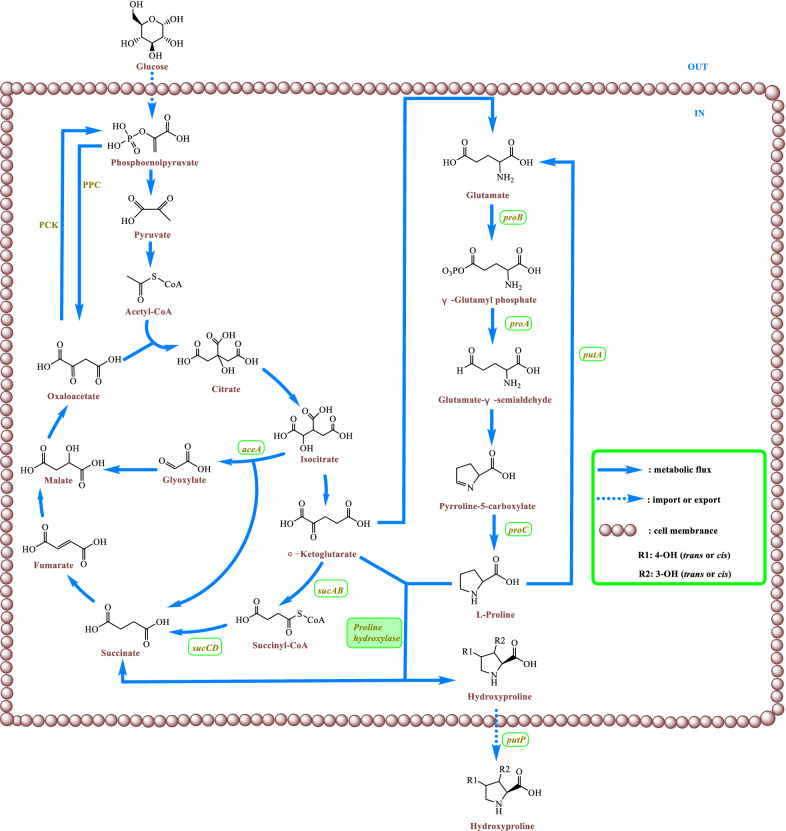
Biosynthetic pathway of hydroxyproline

**Fig. 4:**
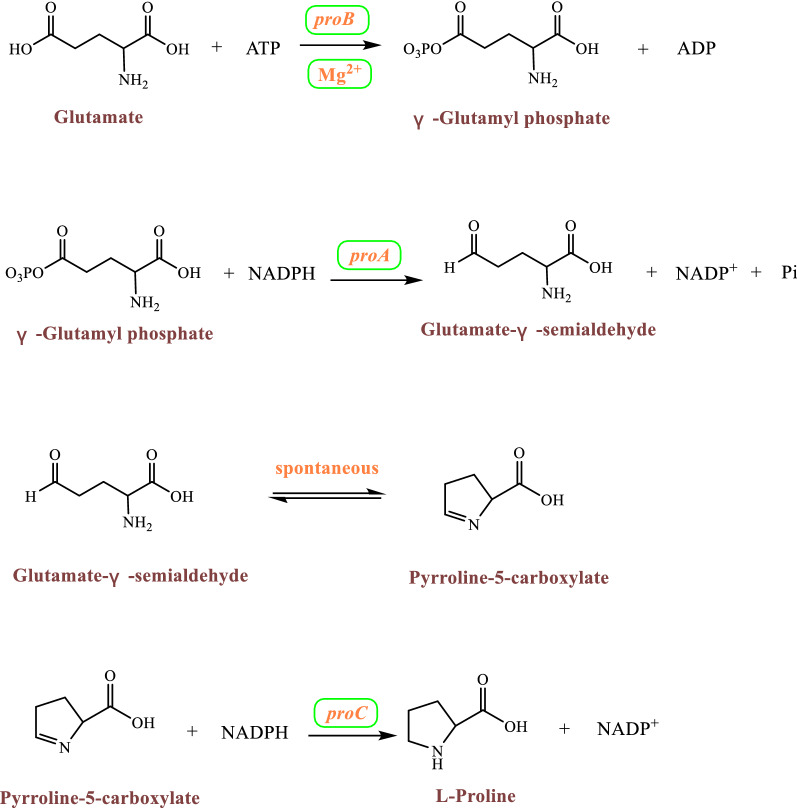
Synthesis pathway from glutamate to proline

The main types of microorganisms currently used to produce *t*4Hyp are *Escherichia coli* and *Corynebacterium glutamicum* (Fig. 3) [19]. In particular, because of a lack of proline hydroxylases, proline hydroxylase is introduced into *E. coli* and *C. glutamicum* and exogenously expressed to form a complete *t*4Hyp synthesis pathway. Glucose as a substrate undergoes a series of biochemical reactions to produce α-ketoglutarate (α-KG). Subsequently, α-KG is catalyzed to form glutamate via glutamate synthase. The synthesis pathway from glutamate to proline requires three enzymatic reactions (Fig. 4) [20]. First, glutamate is catalyzed to γ-glutamyl phosphate by γ-glutamyl kinase. This enzyme is the rate-limiting enzyme of proline synthesis and can be inhibited by proline feedback [21]. Typically, the enzyme is a homotetramer of a 367-amino acid polypeptide comprising an N-terminal 257-residue amino acid kinase (AAK) domain along with a 110-residue PUA (named for pseudo uridine synthases and archaeosine-specific transglycosylases) domain [22]. Glutamate-γ-semialdehyde dehydrogenase catalyzes the second reaction of proline biosynthesis and activates γ-glutamyl phosphate to glutamate-γ-semialdehyde. The enzyme is not susceptible to either end-product prohibition or inhibition by proline [23]. γ-Glutamyl kinase and glutamate-γ-semialdehyde dehydrogenase occur in a complex, and γ-glutamyl kinase activity is undetectable in the absence of glutamate-γ-semialdehyde dehydrogenase [24, 25]. Glutamate-γ-semialdehyde then cyclizes spontaneously to form pyrroline-5-carboxylate (an internal Schiff base). In the third reaction of the proline biosynthetic pathway, pyrroline-5-carboxylate is reduced to proline by pyrroline-5-carboxylate reductase. Pyrroline-5-carboxylate reductase mediates the pyridine nucleotide-linked reduction of pyrroline-5-carboxylate to proline but not the reverse reaction, even at high substrate concentrations. NADPH and NADH are both cofactors of pyrroline-5-carboxylate reductase, and NADPH demonstrates higher activity than NADH [26].

## Catabolism of proline

**Fig. 5:**
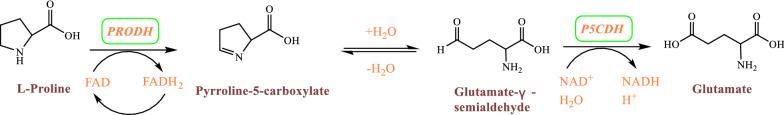
Proline is catabolized to glutamate via two enzymatic steps. *PRODH* proline dehydrogenase, *P5CDH* pyrroline-5-carboxylate dehydrogenase

Proline can be catabolized to glutamate through two enzymatic steps (Fig. 5). The *E. coli* PutA protein comprises 1320 amino acid residues, and its dimer has a molecular mass of 293 kDa [27]. PutA is a multifunctional flavor-protein that is both a membrane-associated proline catabolic enzyme and a transcriptional repressor of a pair of genes encoding proline utilization proteins [28]. The first enzymatic step is that proline is catalyzed to generate pyrroline-5-carboxylate by PutA. The electrons from reduced FAD are subsequently transferred to an acceptor in the electron transport chain to complete the catalytic cycle. In the second step of reaction, non-enzymatic hydrolysis of pyrroline-5-carboxylate generates glutamic semi-aldehyde; subsequently, glutamic semi-aldehyde is oxidized to glutamate through the transfer of two electrons to reduce NAD^+^ to NADH [29]. There is some evidence that PutA has DNA-binding activity and serves as a transcriptional repressor of the *putP* and *putA* genes (proline utilization regulon) in *E. coli* [27]. In *E. coli*, the regulation of *put* genes relies on the concentration of proline and intracellular location of the PutA protein [30]. In the presence of proline, the localization of PutA changes from the cytoplasm to the membrane. The transcription of *put* genes is then activated, and proline is oxidized to glutamate [31]. In the absence of proline, PutA accumulates in the cytoplasm and inhibits the transcription of the *put* genes through binding to promoter sequences in the intergenic DNA region [32].

## Proline hydroxylases

**Fig. 6:**
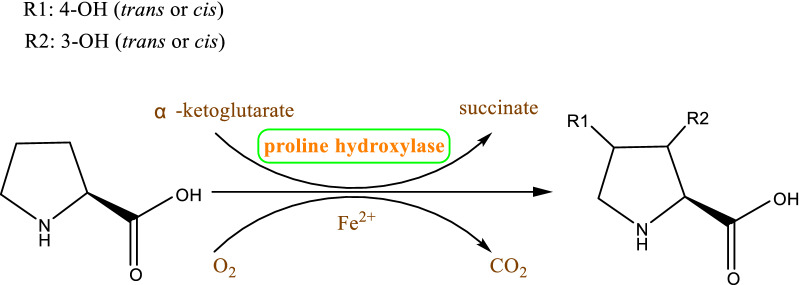
Hydroxylation of proline hydroxylase requires proline, with α-ketoglutarate, Fe^2+^, and O_2_ as cosubstrates

**Fig. 7:**
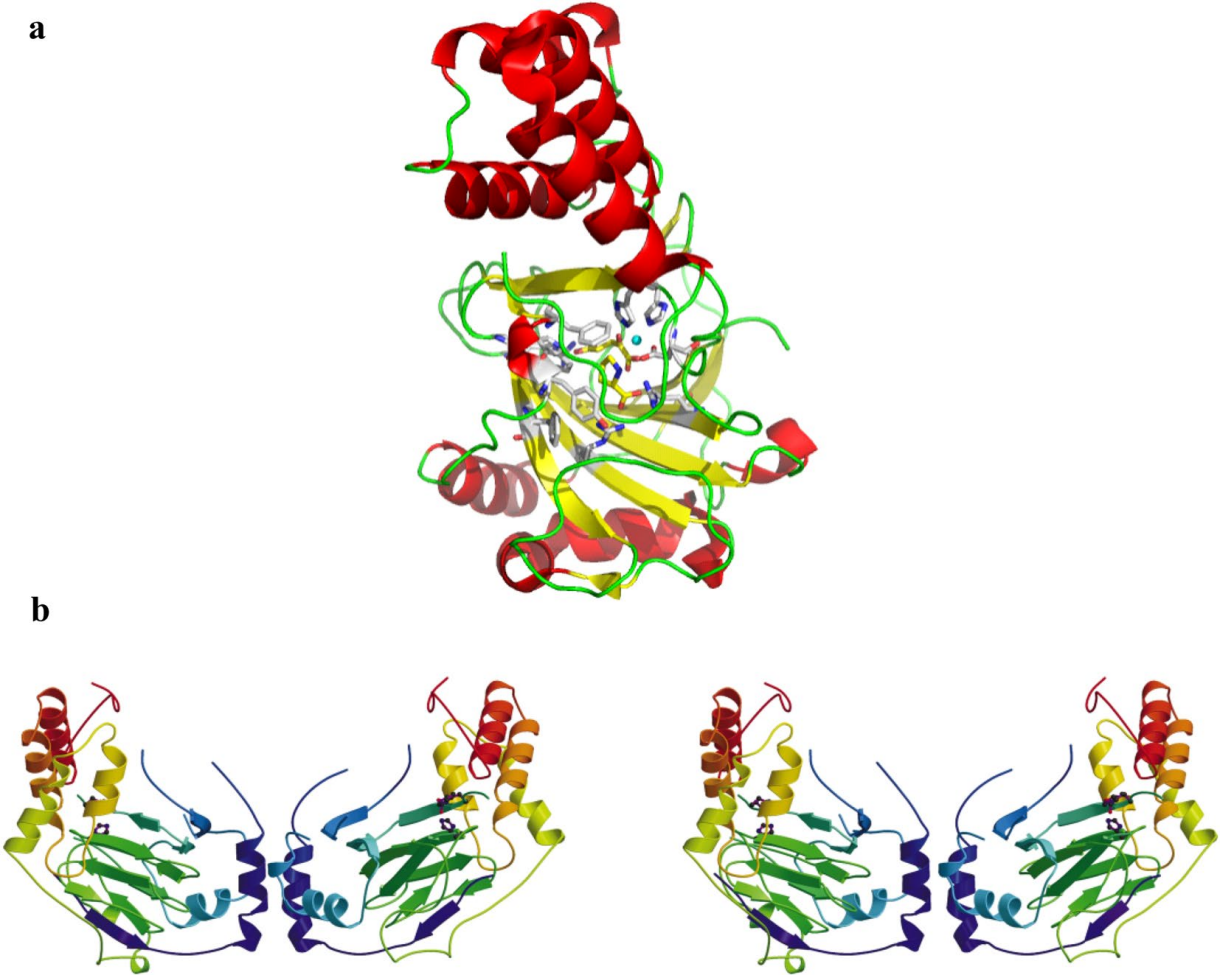
Protein structure. **a** l-proline *cis*-4-hydroxylase. **b** l-Proline *cis*-3-hydroxylase shown in stereo, with the dimer formed by the A and B molecules

**Fig. 8:**
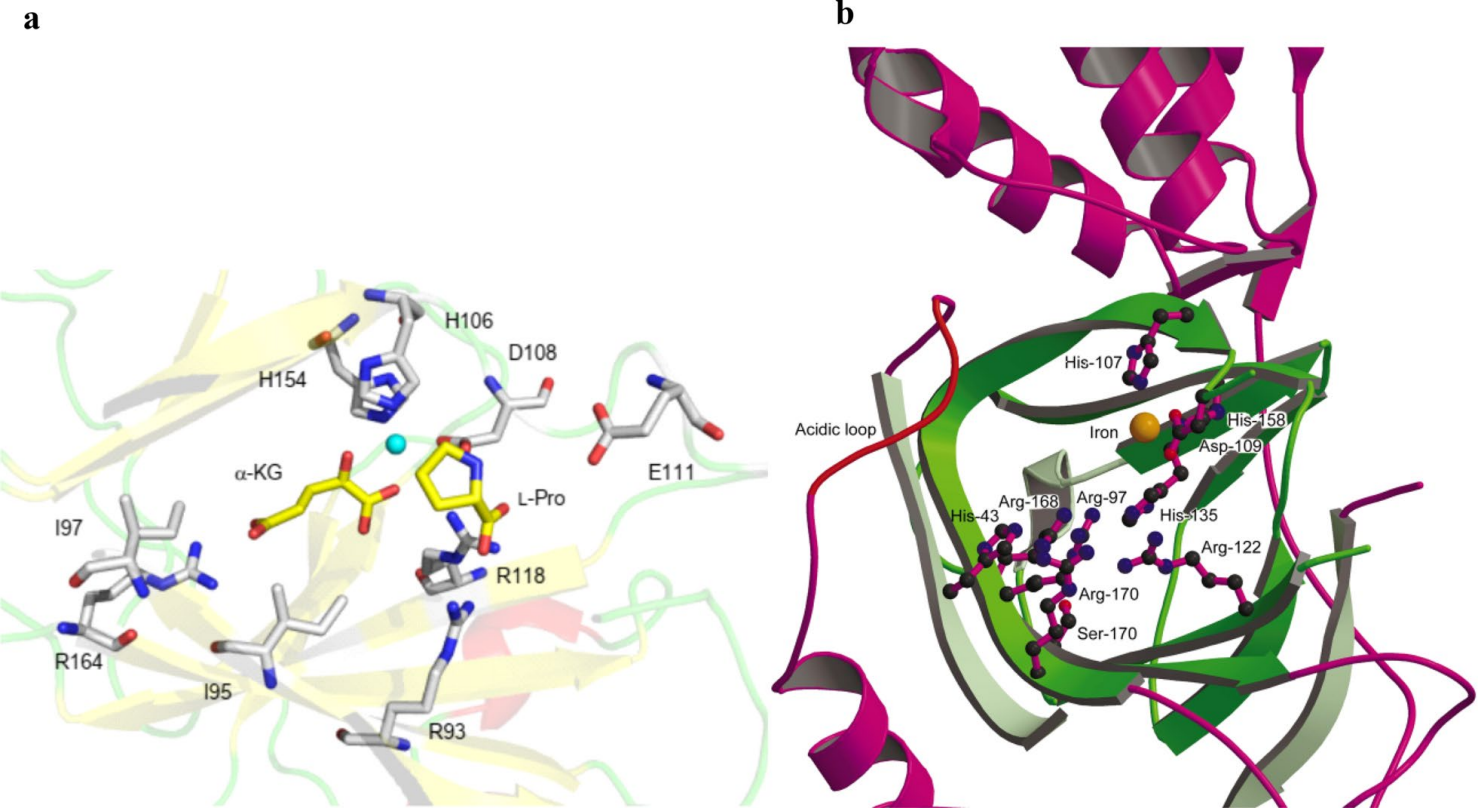
View of the active site. **a** l-proline *cis*-4-hydroxylase. **b** l-Proline *cis*-3-hydroxylase

In mammalian systems, prolyl hydroxylases hydroxylate L-proline residues to Hyp residues, which are involved in collagen biosynthesis, through posttranslational modification of procollagen [33]. The prolyl hydroxylases can be as oxygen sensors, due to the sensitivity to graded levels of oxygen with a strikingly low O_2_ affinity (a Km of 178 mm Hg), which is above the concentration of dissolved O_2_ in the air) and obvious effects of small changes in oxygen concentration on enzymatic activity were observed [34–36]. However, these prolyl hydroxylases accept peptidyl proline, not free l-proline, as a substrate. By contrast, in microbial system, proline hydroxylases exclusively convert free proline to Hyp [37]. Additionally, proline hydroxylases require α-KG and O_2_ as co-substrates and Fe^2+^ as a cofactor, and they are also called Fe(II)/α-ketoglutarate-dependent hydroxylases(Fig. 6) [38]. Proline hydroxylases from microorganisms usually participate in the modification of proline and biosynthesis of antibiotics [39]. To date, four different types of l-proline hydroxylases have been identified in microorganisms, l-proline *trans*-3-hydroxylase (*trans*-P3H, from an uncultured bacterium esnapd13 and *Glarea lozoyensis*) [40, 41], l-proline *trans*-4-hydroxylase (*trans*-P4H, from *Dactylosporangium* sp.RH1) [42], l-proline *cis*-3-hydroxylase (*cis*-P3H, from the *Streptomyces* sp. strain TH1) [43], and l-proline *cis*-4-hydroxylase (*cis*-P4H, from *Mesorhizobium loti* and *Sinorhizobium meliloti*) [44], and convert l-proline to generate *trans*-3-hydroxy-l-proline, *trans*-4-hydroxy-l-proline, *cis*-3-hydroxy-l-proline, and *cis*-3-hydroxy-l-proline, respectively. Among these hydroxylases, *trans*-P4H has been used in large-scale industrial applications of *trans*-4-hydroxy-l-proline [38]. Thus far, the structure of *trans*-P4Hs remains to be elucidated, while the overall structure of *cis*-P4H from *Mesorhizobium loti* and *cis*-P3H from *Streptomyces* sp. TH1 were solved (Fig. 7). The active site comprised a twisted jelly roll β-sheet core, sandwiched by the N-terminal and C-terminal α-helical domains (Fig. 8) [45]. The characteristics of these hydroxylases should be noted, including the kinetic parameters, influence of catalase, ascorbate, α-KG substrate, ferrous iron, EDTA, metal salts, organic acids, pH, and temperature. Based on the observation of the decrease of the substrates, The apparent Km of *trans*-P4H from *Dactylosporangium* sp.RH1 is calculated as 0.2 s^−1^ mM^−1^ [46]. Interesting, different research results were obtained about the effects of catalase and ascorbate on hydroxylation, the relative mechanism needed further study. For 2-oxoacid-dependent dioxygenase-mediated reactions, the stimulation of P4H was realized by catalase via scavenging of detrimental peroxide and for many of the 2-oxoacid-dependent dioxygenase family, ascorbate is necessary to obtain a high rate of catalysis [47, 48]. Onishi et al*.* found that catalase does not stimulate the turnover of l-proline; however, ascorbate can activate P4H, which is isolated from *S. griseoviridus* P8648 [37]. By contrast, Lawrence et al*.* found that 0.2–1 mg/mL of catalase causes a 20–30% increase in the turnover of l-proline, whereas 1 mM ascorbate has a toxic effect because ascorbate competes for the same binding site between α-KG and hydroxylase [49]. However, superfluous ascorbate cannot inhibit the activity of hydroxylase in the *Dactylosporangium* sp. strain RH1 [42]. Mori et al*.* suggested that ascorbate is not necessary for the hydroxylation reaction but can accelerate hydroxylation. α-KG and ferrous ions are strictly required for hydroxylases [43]. If α-KG is replaced with 0.5 mM 2-oxopentanoate, 2-oxoadipate, pyruvate or 2-oxomalonate, l-proline hydroxylation does not occur [49]. These results indicate that hydroxylases belong to the α-ketoglutarate-dependent dioxygenase family. The addition of EDTA (a chelator of divalent cations) and divalent cations (Zn^2+^, Cu^2+^, Ni^2+^, Co^2+^, Mg^2+^, and Mn^2+^) strongly inhibits enzyme activity [41, 43, 44], indicating that these metal ions act as competitive inhibitors with Fe^2+^ and that this sensitivity is a common characteristic of members of the α-ketoglutarate-dependent dioxygenase family. The addition of citric acid inhibits the hydroxylation reaction; however, succinate does not inhibit this reaction [43]. The effects of pH and temperature on hydroxylases were also investigated. Maximal activities were achieved at a pH and temperature of approximately 7.0 and 25 °C, respectively [41, 44]. An improvement in hydroxylase-specific activity was observed when proline is supplied to the medium compared with when the medium is not supplemented, indicating an induction effect of proline [41].

## Metabolic engineering approaches for *trans*-4-hydroxy-l-proline production (Table 1)

**Table 1:** Overview of metabolic engineering studies on the production of *trans*-4-hydroxy-l-proline by microbial cells

Host strain	Expression vectors	Metabolic engineering strategies	Titer (g/L)	Culture system	References
*E. coli*	pTr2-4OH	(1) + *Da-p4h*^o^; (2) promoters^o^; and (3)Δ*putA*;	41	Batch, 5 L (glucose + proline)	[58]
*E. coli*	pSTV29	(1) + *Da-p4h*; (2) + proB*; (3) + *proA*; and (4)Δ*putA*	25	Batch, 5 L (glucose)	[38]
*E. coli* and *C. glutamicum*	pET28a	Compared with the *Ps-p4h* and *Bo-p4h*, and *Da-p4h*	2.28 or 6.72	Batch, 250 mL(glucose) or Batch, 250 mL (glucose + proline)	[19]
*C. glutamicum*	pEKEx2	+ *p4h*	7.1	Fed-batch, 3 L (glucose + isoleucine)	[70]
*E. coli*	pUC19	(1) + *Da-p4h*^o^; (2) expression vectors^o^;	25.4	Fed-batch, 5 L (glycerol + proline)	[81]
*E. coli*	pTrc99a	(1) + *Da-p4h*; and (2) + VHb	14.4	Fed-batch, 1.4 L (glucose + proline)	[75]
*E. coli*	pTrc99a	(1) + *Da-p4h*; (2)Δ*sucAB*; (3)Δ*aceAK*; (4)Δ*putA*; and (5) + proB*; and (6) + *proA* gene	31	Fed-batch, 5 L (glucose)	[67]
*E. coli*	pTc	(1) + *p4h*^gm^; (2)Δ*putA*; (3) + proB*; and (4) + *proA*	45.83	Fed-batch, 5 L (glucose)	[55]
*C. glutamicum*	pXMJ19	(1) + *Da-p4h*; (2)Δ*sucCD*; and (3) fine-tuning of proB* and *p4h* abundances	21.72	Fed-batch, 500 mL (glucose)	[62]
*E. coli*	pET21a	(1) + *Un-p4h*; (2) + *p4h*^re^; (3) p4h*; (4)Δ*putA*; (5) + proB*; and (6) + *proA*	12.9	Fed-batch, 5 L (glucose)	[57]
*E. coli*	pDXW-10	(1) + *p4h*^*rd*^; (2) + *p4h*^o^; (3)Δ*putA*; (4)Δ*proP*; (5)Δ*putP*; (6)Δ*aceA*; (7) + proB*; and (8) a tunable circuit based on quorum sensing	54.8	Fed-batch, 7.5 L (glucose)	[69]

^o^: optimization of the gene codon usage or promoters or expression vectors; ^gm^: genome mining; ^re^: replacement of the gene putative “lid” loop; ^rd^: rational design; +: expression of the gene; Δ: deletion of the gene; *: mutation of gene; *Da*: *Dactylosporangium* sp*.*RH1; *Ps*: *Pseudomonas stutzeri*; *Bo*: *Bordatella bronchiseptica RB50*; *Un*: uncultured bacterium esnapd13

*T*4Hyp can be produced in different ways (e.g., chemical synthesis or extraction), but these methods are associated with high cost, environmental pollution, and low productivity [50, 51]. Thus, *t*4Hyp production by metabolic engineering may be a competitive approach. Thus far, *t*4Hyp production by microorganisms has been widely studied.

## Optimization, genome mining and evolution of hydroxylases

The crucial factors for converting l-proline to *t*4Hyp are hydroxylases, which are promising targets to improve the hydroxylation efficiency to obtain a higher *t*4Hyp titer and productivity. Because the hydroxylase gene from the *Dactylosporangium* sp. strain RH1 has a high GC content and induces rare codons in *E. coli*, it is poorly expressed in *E. coli*. The 5′ end of the proline 4-hydroxylase gene was optimized by cassette mutagenesis according to the codon usage of *E. coli* genes. The optimized proline 4-hydroxylase gene ligated into the plasmid had 7.7-fold higher activity than the original gene [51]. However, if the codon is only designed to achieve the highest abundance, the tRNAs charged with amino acids that are highly represented may be depleted, and translational errors in the heterologous protein may occur. A special codon optimization strategy that stimulates the relative codon usage of *E. coli* was proposed to verify that proline 4-hydroxylase production is limited by the charged tRNAs. This method increased proline 4-hydroxylase levels by two to fourfold. Furthermore, because the mRNA levels of the optimized gene generated by the special codon optimization strategy were tenfold higher than those observed for the original gene, the increase in proline 4-hydroxylase synthesis may also have been related to mRNA stability. Another experiment also confirmed that codon usage has a large impact on proline 4-hydroxylase synthesis and that the optimized gene produced higher proline 4-hydroxylase levels in the absence of proline than the original gene in the presence of proline [51]. A high amount of insoluble protein in *E. coli* may be present when a gene is expressed via commercial vectors. However, in most cases, the active protein tends to be soluble. To obtain much larger amounts of soluble protein, Chen et al*.* constructed 12 truncated variants of the proline 4-hydroxylase gene by predicting the secondary structure of proline 4-hydroxylase and removing some of the N-terminal or C-terminal amino acids, including the 1–240, 1–257, 1–272, 5–240, 5–257, 5–272, 15–240, 15–257, 15–272, 25–240, 25–257, and 25–272 truncated fragments. Compared with the full-length protein composed of 272 amino acids, the 1–257 truncated fragment generated much more soluble protein and had a higher catalysis efficiency (conversion: 88.97% in 60 h) [52]. The hydroxylase gene used to produce *t*4Hyp from *Dactylosporangium* sp. RH1, which has good catalytic activity, can be called *p4hD*. However, if used in industrial production, higher activity is required. Thus, hydroxylases need to be improved or new hydroxylases need to be isolated. The P4H proteins from *Pseudomonas stutzeri* and *Bordetella bronchiseptica RB50*, named *p4hP* and *p4hB*, respectively, were isolated and characterized, and they were found to exhibit lower catalysis activity than the *p4hD* gene of P4H [53]. In the past few decades, genome mining has proven to be an effective method to discover new enzymes [54]. Using the genome mining approach to search for new hydroxylases from the GeneBank database, Wang et al*.* found 3 novel *trans*-proline-4-hydroxylases from *Alteromonas mediterranea* (AlP4H), *Micromonospora* sp. CNB394(MiP4H) and *Sorangium cellulosum* (ScP4H) from 101 candidates and performed enzymatic determination. AlP4H exhibited the highest *t*4Hyp production among the *trans*-proline-4-hydroxylases, including the hydroxylase from *Dactylosporangium* sp. RH1 [55]. A better understanding of this family of α-ketoglutarate-dependent dioxygenases is necessary. The 3D structure of a leucine hydroxylase from *Streptomyces* DSM 40835 has been characterized, and a loop region (K159-S176) in the leucine hydroxylase covers its active site and plays an important role in substrate binding and access [56]. Compared with the structures of leucine hydroxylase and *trans*-proline-4-hydroxylases, replacing the lid loop may alter its enzymatic properties. Liu et al*.* replaced a new *trans*-proline-4-hydroxylase from the uncultured bacterium esnapd13 (UbP4H) putative “lid” loop in combination with site-directed mutagenesis to enhance hydroxylase activity and thermos-stability [57].

## Optimization of expression elements

To improve *t*4Hyp production by regulating the expression levels of the genes and harmonizing the metabolic pathways in the host system, optimization of expression elements is required. The *trp*, *trp* tandem, and *tac* promoters were evaluated. Among them, the *trp* tandem promoter showed higher activity than the other promoters [58]. Regarding vectors, pET-M-3C containing a His fusion tag and the T7 promoter, pET-32M-3C containing a Trx-His fusion tag and the T7 promoter, pUC18 containing no fusion tag and the *lac* promoter, and pTTQ18 containing no fusion tag and the *tac* promoter were evaluated. pET-M-3C showed a higher substrate conversion efficiency (conversion: 76.60% in 60 h) than the other vectors [52]. These results suggest that the expression system exerted an obvious effect on enzyme activity and *t*4Hyp production. An important process is the regulation of the initial translation rate of the protein and determination of its protein abundance, which involves the interactions of ribosomes with the 5′ untranslated regions and mRNA folding energetics. To accelerate protein synthesis and increase protein abundance, the rate of ribosome binding to mRNA should be faster than that of the refolding of mRNA [59, 60]. The ribosome binding site (RBS) can be optimized using the software RBS Calculator v2.0 to achieve the maximum translation initiation rate [61]. The optimized RBS and *p4h* gene should be co-expressed in an engineered strain. As expected, the abundance of P4H was higher than that of the original gene, and the new strain produced up to 8.47 g/L of *t*4Hyp, which was increased by 76% compared with that in the original strain. Moreover, the maximal specific P4H activity and productivity also increased by 67% and 68%, respectively [62]. The same result was observed because the optimization of the RBS strength improved the balance of *t*4Hyp production and cell growth to benefit and enhance *t*4Hyp productivity [63].

## Deletion of the proline degradation pathway

As mentioned above, proline can be degraded into glutamate via the PutA protein. To examine the effect of proline metabolism and its regulation on proline hydroxylation, how the *putA* gene affects *t*4hyp synthesis should be investigated. Shibasaki et al*.* deleted the *putA* gene of *E. coli* to block the pathway from proline to glutamate and increased the yield of *t*4Hyp from proline (87 to 100%) [58]. Additionally, the concentration of glutamate was decreased when the *putA* gene was deleted, while *t*4Hyp production and its conversion rate were increased by 35.0%, up to 41.3 g/L and 72.5%, respectively [63]. Although the Δ*putA* strain could not degrade proline, reducing energy consumption in response to low glucose uptake rates to offset the loss of ATP and NADH did not increase TCA activity [64]. These results suggest that proline degradation is one of the limited factors affecting *t*4Hyp production and that the deletion of the proline degradation pathway is a promising strategy to improve the hydroxylation efficiency.

## Regulation of proline availability

In the proline biosynthetic pathway, proline synthesis from glutamate is sequentially catalyzed by *proB* (encoding γ‐glutamyl kinase), *proA* (encoding γ‐glutamyl phosphate reductase) and *proC* (encoding pyrroline‐5‐carboxylate reductase). The regulatory mechanism of the proline biosynthetic pathway is the feedback inhibition of ProB by proline. ProB74, a ProB mutant, showed a 360-fold lower sensitivity to feedback inhibition by proline [20]. When ProB74 was used to produce *t*4Hyp, the productivity was much higher than that of ProB [38, 65]. Considering that L-proline is an important precursor of *t*4Hyp and may be a limiting factor for *t*4Hyp production, the availability of l‐proline for hydroxylation should be regulated. Given that the gene expression levels of the *proB*74 and *p4h* genes might affect the proline formation and proline hydroxylation rates, Zhang et al. designed two expression cassettes, P*tac*-*p4h*-*proB*74 and P*tac*-*proB*74-*p4h*, as well as an optimized RBS and universal RBS that were separately used to control the protein levels of the *proB*74 and *p4h* genes. A higher translation initiation rate of *proB*74 contributed to an increase in carbon flux for proline synthesis. However, with the same translation initiation rate of proline 4-hydroxylase, only a low protein level of ProB74 improves the conversion efficiency from proline to *t*4Hyp, and the engineered strain produces up to 21.72 g/L *t*4Hyp with a yield of 0.27 mol/mol and a productivity of 0.36 g/L/h [62]. From the above data, a higher translation initiation rate of hydroxylase combined with a lower protein level of feedback-resistant γ-glutamyl kinase contributes to increasing the hydroxylation efficiency.

## Reconstruction of the tricarboxylic acid cycle (TCA) and glyoxylate pathway

The TCA cycle is a key metabolic pathway that connects carbohydrate, fat, and protein metabolism. Because *a*-ketoglutarate (α-KG) serves as an intermediate of the TCA cycle and hydroxylases, the availability of α-KG has an important effect on *t*4Hyp production, while proline hydroxylases can convert cosubstrates (including α-KG) to succinate. Thus, cell growth competes with proline hydroxylation. Additionally, α-KG can be shunted for glyoxylate synthesis. To force and improve α-KG flux to the proline hydroxylation reaction, reconstruction of the TCA cycle and glyoxylate pathway should be performed. The TCA cycle involves several key metabolic genes, such as *sucAB*, which encodes α-ketoglutarate dehydrogenase, and *sucCD*, which encodes sucCoA synthetase [65]. In the glyoxylate pathway, *aceA* encodes isocitrate lyase (AceA) and *aceK* encodes isocitrate dehydrogenase kinase/phosphatase (AceK). The influence of the modification strategy on *t*4Hyp production depends on which genes should be modified. When evaluating the influence of α**-**KG dehydrogenase or sucCoA synthetase deletion on host physiology, the *SucA* (α-KG dehydrogenase E1 subunit) and *sucC* (sucCoA synthetase β subunit) genes were alternately deleted in combination with *aceA* and *putA*. Severe impairment of sucCoA synthesis was observed; for example, the triple mutant strain 3Δ*sucA* (Δ*sucA*Δ*aceA*Δ*putA*) could not grow in minimal medium; however, the introduction of proline 4-hydroxylase utilizing proline hydroxylation as a TCA cycle bypass restored growth, causing a lower growth rate, final biomass concentration, and average cell size than those of the wild-type strain due to the decreased sucCoA pool and deficiency of the glyoxylate pathway. By contrast, no obvious effect was observed for the triple mutant strain 3Δ*sucC* (Δ*sucC*Δ*aceA*Δ*putA*), which could grow in minimal medium. These results indicate that the *sucC* deletion strain obtained sucCoA from α**-**KG via α**-**KG dehydrogenase [66]. Similar results were observed because the deletion of *sucAB* and *aceAK* in *E. coli* inhibits the pathways of succinate biosynthesis, glyoxylate, and IDH phosphorylation, which contribute to coupling l-proline hydroxylation with strain growth. Thus, the modified strain exhibited higher conversion of l-proline and a higher yield of *t*4Hyp [67]. To improve *t*4Hyp production, a genome-scale metabolic network model was applied to identify potential targets. Zhang et al. set *t*4Hyp production as the maximal object, and the biomass and flux via α‐ketoglutarate dehydrogenase and succinyl-CoA synthetase dropped to zero. However, when the biomass was set as the maximal object, zero flux via succinyl-CoA synthetase and low flux via α‐ketoglutarate dehydrogenase increased *t*4Hyp production. Thus, these in silico simulation results indicate that the deletion of *sucCD* genes encoding succinyl-CoA synthetase may improve *t*4Hyp production. Consequently, the Δ*sucCD* strain produced 4.81 g/L of *trans*‐4‐hydroxy-l-proline, 60% higher than the amount produced by the wild-type strain. Furthermore, the yield of *trans*‐4‐hydroxy-l-proline on glucose increased by 50% [62]. This finding was confirmed by simultaneously deleting the *sucC* and *sucD* genes in *E. coli*; the resulting strain, *E. coli* Δ*sucCD*, showed a 139.1% increase in *t*4Hyp production up to 30.6 g/L without α-KG addition compared with that of the wild-type strain [63]. In *Corynebacterium glutamicum*, the activity of the α-ketoglutarate dehydrogenase complex (ODHC) can be dynamically modulated by l-isoleucine-responsive transcription or attenuation strategies [68]. A similar strategy was adopted to regulate ODHC activity in *E. coli*, in which a tunable circuit based on quorum sensing was used to improve the flux of α-KG by twisting the flow between the TCA cycle and *t*4Hyp production; this approach also showed better performance than that achieved by directly deleting *sucA* [69].

## Improvement of oxygen transfer

Given the catalytic characteristics of proline hydroxylase, large amounts of oxygen are required for *t*4Hyp production [42]. During fermentation, the soluble protein or other substance secreted by the strain will cause the fermentation broth to possess a high viscosity, which interferes with oxygen transfer and decreases the dissolved oxygen levels. Thus, the oxygen supply is usually considered a rate-limiting factor for *t*4Hyp production [70]. To solve this problem, several strategies may be attempted: (1) increasing the agitation, aeration rates, and pressure of the bioreactor, (2) introducing the *Vitreoscilla* hemoglobin gene (*vgb*), or (3) adding a protease. However, increasing the agitation and aeration rates may cause extensive energy consumption and physical damage to the cells [71]. Bacterial hemoglobin (VHb) is necessary for *Vitreoscilla,* a strict aerobic bacterium, to survive in an oxygen-poor environment [72]. In high-cell-density fermentation processes, the presence of VHb enhances ATP production and energy metabolism by promoting oxygen transfer under O_2_-limited conditions, which has been widely used to improve the growth and production of target products [73, 74]. Zhao et al. integrated the *Vitreoscilla* hemoglobin gene (*vgb*) into the chromosome of recombinant *E. coli* to increase *t*4Hyp production. In a shaking flask culture, the strain expressing *Vitreoscilla* hemoglobin (VHb) produced 8.23 g/L of *t*4Hyp, which was approximately a 1.9-fold increase over that of the strain not expressing VHb (4.24 g/L) [75]. Additionally, in a 5-L fermenter, *t*4Hyp production (48.8 g/L) was increased by 2.5% than that in the absence of VHb overexpression, and the productivity (1.02 g/L^/^h) was increased by 54.5% [63].

## *Trans*-4-hydroxy-l-proline production in *Corynebacterium glutamicum*

To date, research on *t*4Hyp production has mainly focused on *E. coli*. However, *Corynebacterium glutamicum*, a nonpathogenic, Gram-positive bacterium, which is a popular industrial microorganism and is usually applied in amino acid, vitamin and nucleic acid production, should be introduced for *t*4Hyp production [76]. In 1957, this bacterium was first discovered for its excellent ability to produce abundant l-glutamate [77]. *C. glutamicum* has been studied thoroughly for its amino acid production, and several molecular tools for metabolic network operation that benefit *C. glutamicum* as an important host system have been developed [78, 79]. The availability of intracellular proline may be limited because the biosynthesis of proline in *E. coli* is strictly regulated to a low level and the high l‐proline production in *C. glutamicum* makes *C. glutamicum* a better candidate for *t*4Hyp production [80]. A novel strategy applies recombinant *C. glutamicum* strains to produce *t*4Hyp, and the differences between *C. glutamicum* and *E. coli* as hosts for *t*4Hyp production are observed when the hydroxylase genes from diverse resources are cloned and expressed simultaneously. Microbial systems for *t*4Hyp production have been constructed by introducing proline 4-hydroxylase into *C. glutamicum* and *E. coli*. Although both recombinant *C. glutamicum* and *E. coli* strains can produce *t*4Hyp, a higher yield (0.47 g/L) was observed in recombinant *E. coli* than in *C. glutamicum* [53]. To produce *t*4Hyp from glucose in minimal medium, Falcioni et al. introduced the proline 4-hydroxylase gene into an isoleucine-deficient *C. glutamicum* strain. The effects of various glucose/isoleucine molar ratios on *t*4Hyp production were observed, and a molar ratio of 46:1 was found to maximize *t*4Hyp production (0.2 g/L) [70]. Zhang et al. engineered *C. glutamicum* by reconstructing the TCA cycle, expressing the proline 4-hydroxylase gene and fine-tuning the abundances of plasmid-borne feedback-resistant γ-glutamyl kinase and proline 4-hydroxylase for the fermentative production of *t*4Hyp, achieving the highest titer (21.72 g/L) in glucose-minimal medium [62]. Although a large amount of *t*4Hyp production in *C. glutamicum* was not efficiently synthesized, it remains challenging to develop *t*4Hyp‐producing *C. glutamicum* with excellent fermentation performance considering the characteristics of high proline production.

## Metabolic engineering of *trans*-4-hydroxy-l-proline production in a bioreactor

To examine the real ability of *t*4Hyp production, the conditions it is must be observed during cultivation in a bioreactor. The culture conditions (pH, temperature, agitation of the jar fermenter, induction time, and bioreactor type) significantly affect microbial *t*4Hyp production. The pH and temperature used for *t*4Hyp production by *E. coli* are usually 6.0–7.0 and 30–37 °C, respectively. In addition to temperature and pH, the initial concentration of l-proline and agitation of the jar fermenter also needed optimization. Combined with optimized promoters and deletion of the *putA* gene, *t*4Hyp can accumulate to a concentration of 41 g/L (100% yield from l-proline) in 100 h when the cells are cultivated in medium containing glucose and Fe^2+^ [58]. *T*4Hyp can be produced in fermentation broth by supplementing the necessary substrate or utilizing harvested cells to induce l-proline hydroxylation in a reaction system. The maintenance of pH is required during this process. Additionally, a feeding strategy usually improves the production of the product. The fermentative production of *t*4Hyp can be divided into two types based on the addition of proline to the fermentative medium. The addition of proline can increase *t*4Hyp production, but it also increases the cost. Achieving a high titer and *t*4Hyp production without proline addition is attractive. The first reported method to produce *t*4Hyp by fermentation in a 5-L jar fermenter did not involve proline addition through the expression of proline 4-hydroxylase from *Dactylosporangium* sp. RH1, feedback resistant γ‐glutamyl kinase (ProB74), or glutamate-γ-semialdehyde dehydrogenase (ProA) combined with the deletion of PutA engineered *E. coli*, and this method achieved 25 g/L of *t*4Hyp after 96 h of cultivation [38]. Although fermentative production of *t*4Hyp has been achieved, the long culture time required and low titer produced limit its industrial application. In *E. coli*, Zhang et al. overexpressed hydroxylase (P4H), γ-glutamyl kinase and glutamate-semialdehyde dehydrogenase (ProBA) and knocked out *putA*, which encodes proline dehydrogenase (PutA), *sucAB*, which encodes α-ketoglutarate dehydrogenase (SucAB), *aceAK*, which encodes isocitrate lyase(AceA), and isocitrate dehydrogenase kinase/phosphatase (AceK) in the TCA cycle, resulting in 31.0 g/L of *t*4Hyp obtained directly from glucose after 52 h of fermentation in a 5-L fermenter; thus, this method reduced the culture time and increased the titer [67]. In a fed-batch fermentation with a 5-L bioreactor without proline addition, Wang et al. accumulated 45.83 g/L of *t*4Hyp within 36 h by introducing a new proline 4-hydroxylase from *Alteromonas mediterranea* (AlP4H), which was discovered through genome mining a *putA*-deficient *E. coli* strain expressing the *proB74* and *proA* genes [55]. To examine *t*4Hyp production by proline 4-hydroxylase via loop grafting and site-directed mutagenesis, a *t*4Hyp-producing strain was constructed, as previously reported [55]. After introducing the evolutive proline 4-hydroxylase, a fed-batch fermentation in a 5-L bioreactor at 37 °C using glucose as the sole carbon source without proline addition was performed. Although only 12.9 g/L of *t*4Hyp was obtained, this concentration was 3.3-fold higher than that produced by the wild-type proline 4-hydroxylase control and simultaneously improved the activity and thermostability [57]. The highest *t*4Hyp production to date was 54.8 g/L at 60 h using integrated system engineering in *E. coli* without L-proline addition in a 7.5-L bioreactor*,* in which the deletion of *putA*, *proP*, *putP*, and *aceA*, and the mutation of *proB* contributed to l-proline and relieved its feedback inhibition, and a tunable circuit based on quorum sensing for the dynamic regulation of ODHC activity and rationally designed l-proline hydroxylase [69]. *t*4Hyp production using proline 4-hydroxylase with chemo-physical combination mutagenesis, taking glycerol as the carbon source, and optimizing the fermentation medium(tryptone, FeSO_4_, l-proline) with single-factor, Plackett–Burman, steepest ascent, and a central composite design experiment, was achieved at 25.4 g/L of *t*4Hyp [81]. Subsequently, Zhao et al. attained a *t*4Hyp concentration of 14.4 g/L by integrating the *Vitreoscilla* hemoglobin gene (*vgb*) into recombinant *E. coli* expressing proline 4-hydroxylase in fed-batch fermentation in a 1.4-L bioreactor after 57 h of cultivation [75]. *T*4Hyp production from glucose in minimal media by *Corynebacterium glutamicum* has also been reported. Although proline was not added, isoleucine supplementation was necessary because the effect of various glucose/isoleucine molar ratios on *t*4Hyp production was observed. High-cell-density fed-batch fermentation in a 3-L KLF reactor was performed and produced 7.1 g/L of *t*4Hyp from glucose at 23 h by introducing proline 4-hydroxylase from the *Dactylosporangium* sp. strain RH1 into isoleucine-deficient (leaky auxotroph) *C. glutamicum* at a glucose/isoleucine molar ratio of 46:1[70].

## Perspectives

Currently, the construction of microbial cell factories for amino acid production is a promising strategy. Metabolic engineering involves improving the development of microbial strains to produce various chemicals by modification of enzymes, transporters, and metabolic pathways. Thus, *t*4Hyp can be produced on a large scale. In the past few years, *t*4Hyp production by engineered *E. coli* has been significantly improved by metabolic engineering. Overexpression of the endogenous pathways for *t*4Hyp biosynthesis or heterologous genes and elimination of competing pathways, combined with evolutive proline hydroxylases, has enabled engineered *E. coli* to produce over 50 g/L of *t*4Hyp. Considering that the culture time must be shortened, the cost must be reduced, and *t*4Hyp production must be improved for industrial application, much more work is needed.

## The key factors in *t*4Hyp production are proline hydroxylases

Although thousands of microorganisms have been screened, few proline hydroxylases with high enzyme activity have been identified. Thus, it is necessary to identify more microorganisms that can produce *t*4Hyp in nature. However, screening is time-consuming and labor-intensive. As reviewed in this paper, overcoming the problem using tools such as genome mining or directed evolution of proline hydroxylases is also a promising strategy to improve activity and stability. The new proline hydroxylases obtained using a de novo enzyme design and computational models may not be comparable with natural proline hydroxylases, but the predicted structure of proline hydroxylases contribute to our understanding of the properties of hydroxylases.

## Regulation of the host cell metabolic network is also important for *t*4Hyp production

The microbial metabolic network is so large and complex that it is difficult to determine which genes need to be modified. Metabolic simulation using a genome-scale metabolic model or ^13^C metabolic flux analysis can help determine the metabolic network and rational design that are already applied to produce *t*4Hyp [82]. Genome modification approaches such as gene knockout might not be the best methods for blocking some biosynthetic pathways, and downregulating gene expression might be a much better strategy. CRISPR interference (CRISPRi) is a useful technique to reduce the transcription of target genes with up to 1000-fold repression. This modified system achieved an RNA-guided DNA recognition platform by coexpressing a modified Cas9 protein (lacking endonucleolytic activity) with an sgRNA designed (20 bp) a complementary region that yields specific silencing of a gene of interest without off-target effects [83]. This CRISPRi technology was employed to repress the expression of the *putA* gene in *E. coli* for *cis*-4-hydroxy-l-proline production, and the yield was increased [84].

In addition to *t*4Hyp, much progress in improving the production of many other amino acids in engineered strains has been made by applying these strategies. By fully elucidating the *t*4Hyp biosynthetic pathway, these strategies and the conception of new ideas should allow improvements in *t*4Hyp production by metabolic engineering.

## Data Availability

All the data generated or analyzed during this review are included in published article.

## Abbreviations

*t*4Hyp: *Trans-*4-Hydroxy-l-proline
*E. coli*: *Escherichia coli*
*C. glutamicum*: *Corynebacterium glutamicum*
TCA cycle: Tricarboxylic acid cycle
ATP: Adenosine triphosphate
FAD: Flavin adenine dinucleotide
NADH: Reduced form of nicotinamide-adenine dinucleotide
EDTA: Ethylene diamine tetraacetic acid
SDS-PAGE: Sodium dodecyl sulfate-polyacrylamide gel electrophoresis
CRISPRi: CRISPR interference
IPTG: Isopropyl β-d-thiogalactoside
RBS: Ribosome binding site
α-KG: α-Ketoglutarate
